# Use of Radiomics to Predict Adverse Outcomes in Patients with Pulmonary Embolism: A Scoping Review of an Unresolved Clinical Challenge

**DOI:** 10.3390/diagnostics15162022

**Published:** 2025-08-12

**Authors:** Miguel Ángel Casado-Suela, Juan Torres-Macho, Jesús Prada-Alonso, Rodrigo Pastorín-Salis, Ana Martínez de la Casa-Muñoz, Eva Ruiz-Navío, Ana Bustamante-Fermosel, Anabel Franco-Moreno

**Affiliations:** 1 Department of Internal Medicine, Hospital Universitario Infanta Leonor, Gran Via del Este Avenue, 80, 28031 Madrid, Spain; xelele54@gmail.com (M.Á.C.-S.);; 2 Department of Medicine, Universidad Complutense de Madrid, 28040 Madrid, Spain; 3 Horus-ML, 28027 Madrid, Spain; 4 Department of Radiology, Interventional and Vascular Radiology, Hospital Universitario Infanta Leonor, 28031 Madrid, Spain; 5 Hospital Universitario Gregorio Marañón, 28007 Madrid, Spain; 6 Hospital Universitario Infanta Leonor, 28031 Madrid, Spain; 7 Venous Thromboembolism Unit, Hospital Universitario Infanta Leonor, 28031 Madrid, Spain

**Keywords:** adverse outcomes, early mortality, prognosis, pulmonary embolism, radiomic features, radiomics, risk prediction, scoping review

## Abstract

**Background:** Inherent to the challenge of acute pulmonary embolism (APE), the breadth of presentation ranges from asymptomatic pulmonary emboli to sudden death. Risk stratification of patients with APE is mandatory for determining the appropriate therapeutic management approach. However, the optimal clinically most relevant combination of predictors of death remains to be determined. Radiomics is an emerging discipline in medicine that extracts and analyzes quantitative data from medical images using mathematical algorithms. In APE, these data can reveal thrombus characteristics that are not visible to the naked eye, which may help to more accurately identify patients at higher risk of early clinical deterioration or mortality. We conducted a scoping review to explore the current evidence on the prognostic performance of radiomic models in patients with APE. **Methods:** PubMed, Web of Science, EMBASE, and Scopus were searched for studies published between January 2010 and April 2025. Eligible studies evaluated the use of radiomics to predict adverse outcomes in patients with APE. The PROSPERO registration number is CRD420251083318. **Results:** Nine studies were included in this review. There was significant heterogeneity in the methodology for feature selection and model development. Radiomic models demonstrated variable performance across studies. Models that combined radiomic features with clinical data tended to show better predictive accuracy. **Conclusions:** This scoping review underscores the potential of radiomic models, particularly when combined with clinical data, to improve risk stratification in patients with APE.

## 1. Background

Acute pulmonary embolism (APE) is a cardiovascular emergency with high mortality, especially in the short term, surpassed only by myocardial infarction and stroke [1]. Therefore, immediate diagnosis is crucial. In six European countries with a combined population of 454.4 million, the estimated annual incidence of symptomatic venous thromboembolism (VTE) events was 761,697, of which 295,982 (39%) were classified as APE. There were 370,012 VTE-related deaths; among these, 126,145 (34%) were due to fatal APE, and 217,394 (59%) occurred in patients with suspected but undiagnosed APE [2]. Therefore, despite ongoing advances in diagnosis, treatment, and prevention, APE-related mortality rates remain high.

The management of APE varies based on the risk stratification of the patients. Some patients with mild APE may be safely treated in the outpatient setting, while others require advanced supportive care or aggressive therapies. The European Society of Cardiology (ESC) guidelines on treating APE propose combining clinical rules, imaging, and laboratory parameters to build predictive scores, which permit an assessment of APE-related early risk of death [3]. Among clinical rules integrating APE severity, the Pulmonary Embolism Severity Index (PESI) and its simplified version (sPESI) (Table 1) have been most extensively validated [4,5,6]. These scores have demonstrated exemplary performance in identifying APE patients at low risk for 30-day mortality. The combination of the PESI and sPESI scores, right ventricle dysfunction on an echocardiogram or computed tomography pulmonary angiography (CTPA), and high-sensitivity cardiac troponin or brain natriuretic peptide concentrations stratify patients with acute APE in four risk categories for early mortality: high (with hemodynamic instability), intermediate–high, intermediate–low, and low risk. Guidelines recommend systemic thrombolysis for high-risk patients and the standard anticoagulant treatment administered in a hospital or outpatient setting for intermediate- and low-risk patients. The main strength of the PESI and sPESI scores lies in the reliable identification of patients at low risk for 30-day mortality. However, the scores may not adequately identify patients on the high- and intermediate-risk spectrum [7]. In addition, their performance may be suboptimal in specific clinical scenarios excluded in the derivation cohort, such as patients with high embolic burden, pregnant or postpartum, and patients with SARS-CoV-2 infection, where disease-specific factors may influence risk differently. These limitations highlight the need for more nuanced and individualized prognostic tools to support clinical decision-making in APE.

**Figure 1:**
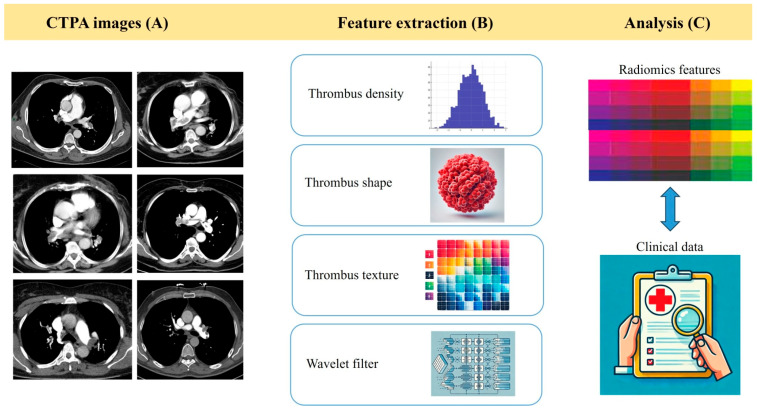
Radiomics study flowchart for outcome prediction in patients with APE. The process begins with thrombus segmentation on contrast-enhanced CTPA (**A**). Multiple radiomic features—such as density, shape, and texture—are extracted from the imaging data (**B**). Subsequently, the data undergo multilevel wavelet decomposition. Finally, the extracted radiomic features may be combined with clinical variables to build machine learning-based prediction models (**C**). Abbreviations: APE, acute pulmonary embolism; CTPA, computed tomography pulmonary angiography.

Radiomics is an innovative, non-invasive approach to imaging, focusing on extracting quantitative features from imperceptible medical images through the visual inspection of traditional radiological practice, thus converting these digital images into high-precision data. Radiomic features capture tissue and lesion characteristics. This technology may be used for clinical problem-solving alone or in combination with clinical, histologic, genomic, or proteomic data. This approach is particularly relevant for survival prediction, as it may support treatment selection and patient risk stratification. For adverse outcome prediction in patients with APE, the hypothesis suggests that a detailed analysis of medical images from CTPA through a radiomic software, which assesses thrombus characteristics such as shape, wave, density, and texture, can accurately identify patients at high risk of early clinical deterioration and mortality. The radiomics study flowchart to predict adverse outcome events in patients with APE is shown in Figure 1. This technique may offer a faster and more precise alternative to traditional clinical scores. A recent editorial analyzed the potential of artificial intelligence (AI) for accurate risk stratification in patients with APE, concluding that AI- and radiomics-based tools hold promises as complementary strategies to improve early risk assessment and support clinical decision-making [8].

The integration of radiomics into the management of APE has emerged as a promising approach to improve risk stratification and predict short-term mortality. This scoping review aimed to analyze the performance of radiomic models for predicting adverse outcomes in APE patients.

## 2. Methods

This review followed the PRISMA 2020 guidelines (Preferred Reporting Items for Systematic Reviews and Meta-Analyses) [9] and adhered to the COSMOS-E recommendations (Conducting Systematic Reviews and Meta-Analyses of Observational Studies of Etiology) [10]. We formulated the research question based on the patient, index test, comparator, outcome, and study design (PICO) criteria as the following: what is the performance of radiomic models (intervention) for predicting outcomes (outcomes) in patients with APE (patients), compared with the current clinical scores (comparator)? The protocol was registered in PROSPERO (International Prospective Register of Systematic Reviews) under ID CRD420251083318.

### 2.1. Literature Search

A comprehensive search was conducted in MEDLINE/PubMed, Web of Science, EMBASE, and Scopus to identify studies on radiomic models predicting adverse outcomes in patients with APE, covering the period from January 2010 to April 2025. The search strategy included the following terms, used as MeSH terms or text words: Artificial Intelligence, Machine Learning, Radiomic, Mortality, Pulmonary Embolism, VTE, CTPA, Outcome, and Prediction. These terms were combined in each database using appropriate headings. The search was restricted to studies involving human subjects, with no language limitations.

### 2.2. Inclusion and Exclusion Criteria

The article’s inclusion criteria were as follows: (a) adult patients, (b) hospitalized or outpatients with APE, (c) the study focused on the use of radiomics to predict adverse outcomes, and (d) PE had to be objectively confirmed by CTPA. The exclusion criteria were as follows: (a) review articles, (b) duplicate publications, and (c) studies without usable data. Cross-sectional studies, case series, and conference abstracts were eligible for inclusion.

### 2.3. Article Selection

Two reviewers (M.Á.C.-S. and A.F.-M.) independently screened titles and abstracts of all retrieved records, followed by full-text articles. Discrepancies were resolved by a third reviewer (J.T.-M.). A standardized screening form was used to support the selection process.

### 2.4. Data Extraction and Analysis

Using a standardized form, data from the studies included were extracted by M.Á.C.-S. and A.F.-M., and reviewed by J.T.-M. The following information was collected: (a) study setting (country, year of publication, and data collection period); (b) study population characteristics (sample size, age, and sex); (c) radiomic software used; (d) outcomes related to APE; and (e) predictive model performance in terms of sensitivity, specificity, negative predictive value (NPV), positive predictive value (PPV), and area under the receiver operating characteristic curve (AUC). According to Swets’ classification, an AUC value between 0.8 and 1.0 was considered adequate [11]. Data pooling was performed using R software, version 4.3.2 (R Foundation for Statistical Computing, Vienna, Austria).

### 2.5. Assessment of Risk of Bias in the Studies

Risk of bias was assessed using the Newcastle–Ottawa Scale (NOS) [12]. The NOS comprises eight items across three domains: selection, comparability, and, depending on study design, either outcome (for cohort studies) or exposure (for case-control studies). Each item includes predefined response options. The study quality is rated using a star system. One star is awarded per item, except for the comparability domain, which allows two stars. The total score ranges from 0 to 9, with scores ≥ 7 considered to be a low risk of bias. Judgments were reached by consensus among three reviewers (M.Á.C.-S., A.F.-M., and J.T.-M.).

## 3. Results

### 3.1. Study Selection

**Figure 2:**
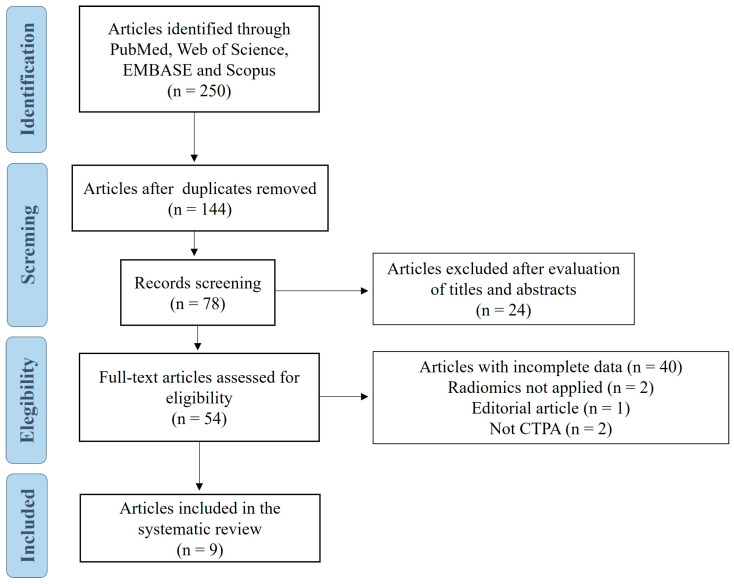
Flow chart of the study selection for the review. Abbreviations: CTPA, computed tomography pulmonary angiography.

Two hundred and fifty studies were initially retrieved, and 144 remained after duplicates were removed. Fifty-four studies were assessed for eligibility with full-text review. Finally, this scoping review included nine original articles (Figure 2) [13,14,15,16,17,18,19,20,21].

### 3.2. Characteristics of the Included Studies

The characteristics of the included studies are summarized in Table 2. Three studies were conducted in China [13,15,21], and six in Germany [14,16,17,18,19,20]. Of note, seven out of the nine studies included in this scoping review were published recently, in 2024 [15,16,17,18,19,20], and one in 2025. All studies had a retrospective design. The studies aimed to predict diverse clinical outcomes related to APE. These outcomes included all-cause mortality at different time points (e.g., 7-day or 30-day), intensive care unit (ICU) admission, need for advanced interventions (such as mechanical ventilation, vasopressor support, or cardiopulmonary resuscitation), and composite clinical deterioration endpoints (Supplementary Table S1). The studies showed significant variability in imaging protocols, including differences in CT scanner models, slice thickness, tube voltage, and contrast administration (Table 3). Radiomic features were extracted from different anatomical regions: seven studies focused on the pulmonary embolus itself [14,15,16,17,18,20,21], one study analyzed epicardial fat [13], and one study analyzed skeletal muscle and intramuscular adipose tissue [19]. MaZda (version 4.6, Technical University of Lodz, Institute of Electronics, Lodz, Poland), ImageJ (version not reported), or PyRadiomics (version 5.1.0) software were used to extract radiomic features from CTPA datasets.

The assessment of the radiomic models is shown in Table 4. The study conducted by Zhou et al. developed a radiomics-based nomogram to predict adverse outcomes in non-high-risk APE patients using cardiovascular parameters extracted from three-dimensional CTPA reconstructions [13]. This study did not analyze the embolus directly but instead extracted morphological indicators of right heart strain from CTPA-derived 3D reconstructions. Specifically, the right-to-left ventricular diameter ratio and interventricular septal curvature were obtained from cardiac chamber reconstructions. Thus, the anatomical localization of extracted features was confined to the heart and interventricular septum, not the thrombus. The ratio of the right ventricular diameter to left ventricular diameter in a four-chamber view/left ventricular diameter in a four-chamber view (RVD4-CH/LVD4-CH) and positive deviation of the interventricular septum curvature were independent predictors of adverse outcomes (OR 7.87 and OR 44.99, respectively), achieving an AUC of 0.87 in the training set and 0.784 in the test set. The model yielded a sensitivity of 50%, specificity of 96.8%, PPV of 76.9%, and NPV of 90.2%. Leonhardi et al. performed texture analysis of pulmonary emboli using 128-slice CTPA images processed with MaZda software [14]. Radiomic analysis was performed on the most proximal visible segment of the pulmonary embolus. Although segmentation methodology was not described in detail, all regions of interest were placed within the contrast-filling defect representing the embolus, without including adjacent vascular structures. They extracted 279 radiomic features and found that parameters such as S (0.5) SumVarnc and S (3,3) SumEntrp were associated with increased mortality and ICU admission (*p* < 0.001), while S (3,−3) AngScMom correlated with sepsis-related organ failure (*p* < 0.001). In a Chinese study, Yang et al. developed a combined model integrating radiomic features and clinical variables (RV/LV ≥ 1.0, age, and sex) to predict 30-day mortality or the need for significant interventions (e.g., thrombolysis, CPR, and mechanical ventilation) [15]. Radiomic features were extracted from manually segmented thrombi identified on CTPA images. The segmentation was performed in 3D Slicer by experienced radiologists, isolating the embolus while avoiding perivascular structures. The resulting regions of interest corresponded anatomically to the entire thrombus volume, with a minimum 2 mm margin from surrounding tissues. Feature extraction using PyRadiomics yielded shape, texture, and filter-transformed metrics, from which a reduced set was selected via LASSO regression. A total of 1037 features were extracted. The combined model achieved an AUC of 0.925 in the training set (sensitivity 93% and specificity 85%) and 0.917 in the validation set (sensitivity 87% and specificity 86%). Gotta et al. (2024a) developed a radiomic model using DECT images and gradient-boosted trees to stratify APE risk [16]. Radiomic features were extracted from contrast-enhanced dual-energy CTPA datasets after semi-automatic segmentation of the pulmonary embolus using the GrowCut algorithm in 3D Slicer (version 4.11.2). The region of interest (ROI) was defined as the contrast-filling defect within the lumen of the central or peripheral pulmonary arteries, as identified on the iodine map series. The segmentation process was manually corrected by experienced radiologists to ensure anatomical precision. The resulting ROI corresponded exclusively to the embolic volume, excluding vessel walls or adjacent structures. Radiomic features were then calculated using PyRadiomics. The model reached an AUC of 0.86 (95% CI 0.645–0.856) in central PE patients and 0.63 (95% CI: 0.38–0.869) in peripheral PE for predicting intermediate–high risk. In another study, Gotta et al. (2024b) evaluated four DECT-based radiomic models for predicting clinical outcomes in APE [17]. Radiomic features were extracted from dual-energy CTPA scans after manual segmentation of the pulmonary embolus using the 3D Slicer software. The segmentation focused on the central thrombus mass as visualized on iodine maps, and the region of interest (ROI) was defined as the intraluminal contrast-filling defect within the pulmonary arteries. No adjacent lung parenchyma, vessel wall, or perivascular structures were included. Segmentation was reviewed and corrected by trained readers to ensure consistency. Feature extraction was performed using PyRadiomics, and only variables derived strictly from the embolic volume were considered for model construction. The unadjusted model achieved an AUC of 0.991; adding age, troponin, or the PESI score did not significantly alter performance, with AUCs ranging from 0.989 to 0.991. Gotta et al. (2024c) also examined the association between thrombus volume, D-dimer levels, and hospitalization in 136 patients [18]. In this study, thrombus segmentation was performed manually using 3D Slicer, with the region of interest (ROI) defined as the contrast-filling defect within the pulmonary arteries visible on CTPA images. The segmentation focused on the entire embolus volume and was reviewed by experienced operators to ensure accuracy. No adjacent vascular walls or lung tissue were included. Radiomic features were extracted from these segmented volumes using PyRadiomics, and the analysis was restricted anatomically to the embolic content. They reported a significant correlation between D-dimer levels and thrombus volume in central PE (*p* = 0.0037), but no association with adverse outcomes (*p* = 0.4719). Their radiomic models had modest performance: the unadjusted model showed an AUC of 0.63, and the adjusted model 0.58. Shahzadi et al. developed radiomic models based on skeletal muscle (SM) and intramuscular adipose tissue (IMAT) to predict 7- and 30-day mortality [19]. They extracted 234 features from a single CTPA slice at T12. In the validation cohort, the combined SM + IMAT model reached an AUC of 0.70 for 30-day mortality (sensitivity 0.74 and specificity 0.54), while sPESI outperformed all radiomic models (AUC 0.74 for 30-day mortality, sensitivity 0.97 and specificity 0.16). Surov et al. investigated the potential of texture analysis in pulmonary emboli to predict short-term survival using contrast-enhanced CT images of 81 patients with acute PE [20]. Radiomic features were extracted with MaZda software, and the study focused on first-order histogram and gray-level co-occurrence matrix (GLCM) parameters. Patients who survived differed significantly from non-survivors in features such as angular second moment (ASM), contrast, correlation, and entropy (*p* < 0.05). Entropy showed the best discriminative power (*p* = 0.001), suggesting that higher heterogeneity within emboli was associated with mortality. Although no predictive model was built, the findings support the potential prognostic role of radiomics. Finally, Wang et al. developed and externally validated three models to predict 30-day all-cause mortality in 224 patients with acute PE undergoing CTPA [21]. Radiomic features were extracted from the embolus region using 3D Slicer with manual segmentation, followed by expert review for margin accuracy. From an initial set of 1316 features, 22 were selected using the LASSO algorithm. The final combined model, which integrated the radiomic score with age, systolic blood pressure, heart rate, NT-proBNP, and troponin I, achieved an AUC of 0.89 (95% CI, 0.83–0.95) in the training cohort and 0.901 (95% CI, 0.808–0.994) in the external validation cohort, outperforming clinical scores such as sPESI and BOVA. The radiomics-only model and the clinical-only model yielded lower AUCs in validation (0.74 and 0.83, respectively). The combined model also showed good calibration and clinical utility in decision curve analysis. Machine learning and deep learning approaches achieved higher AUCs (0.91 and 0.94, respectively) in internal cross-validation but were not externally validated.

### 3.3. Risk of Bias

All studies were rated as having a low risk of bias (Table 5). Most studies fulfilled the criteria for adequate selection of cohorts, appropriate comparability between groups, and reliable outcome assessment.

## 4. Discussion

To the authors’ knowledge, this is the first review specifically addressing the use of radiomics in predicting outcomes in patients with APE. The included studies show that radiomics-based models have a promising capability to predict adverse clinical outcomes, particularly when radiomic features are integrated with clinical variables. This finding supports the potential role of radiomics in improving early risk stratification in APE.

Prognostic information plays a key role in guiding therapeutic decision-making in patients with acute symptomatic APE, such as determining the need for intensive care, escalation of treatment, or thrombolytic therapy. Conversely, accurate prediction models may help identify patients eligible for early discharge and outpatient management. Current European Society of Cardiology (ESC) guidelines recommend using the PESI and sPESI scores to estimate early mortality risk in APE patients [3]. These scores strongly correlate with short-term mortality in external validation studies [4,5,6,22]. However, they present certain limitations: the original PESI score includes 11 weighted variables, which can be impractical in emergencies. At the same time, both PESI and sPESI may underestimate risk in specific subgroups such as younger patients or those with chronic comorbidities.

Radiomics transforms medical images into high-dimensional quantitative data using artificial intelligence and machine learning. A previous systematic review focused on AI-based models for APE detection using CTPA, with pooled sensitivity and specificity values of 88% and 86%, respectively [23]. Another study explored an AI-based model for risk stratification using clinical and laboratory variables, achieving high accuracy (99.2%) and sensitivity (98.5%) [24]. However, these models still require external validation before widespread adoption.

Recent research has explored radiomics not only for diagnosis but also for prognostic purposes in APE. Several studies in this review developed models focused on thrombus-based features, demonstrating good predictive accuracy for identifying patients at high risk of adverse outcomes. Of note, one of the studies by Gotta et al. yielded modest performance when targeting hospitalization risk rather than mortality or severe complications [18].

Two particularly innovative approaches were also identified. Shahzadi et al. developed radiomic models based on skeletal muscle (SM) and intramuscular adipose tissue (IMAT) features extracted from CTPA images at the T12 level to estimate 7- and 30-day mortality [19]. Although the performance of these models was lower than that of the sPESI score in the validation cohort, the study represents an essential step toward incorporating body composition into prognostic assessments. Similarly, Surov et al. investigated epicardial adipose tissue (EAT) texture as a predictor of short-term mortality [20]. Their findings showed that higher heterogeneity in EAT, especially increased entropy, was significantly associated with worse outcomes. These two studies reveal a growing trend in evaluating extrapulmonary structures on routine CTPA scans, suggesting that radiomics may offer a broader, more holistic approach to risk stratification in APE.

In most studies, combining radiomic features with clinical variables—such as age, sex, biomarkers, or PESI score—improved predictive performance, with AUCs reaching up to 0.97 in validation cohorts. A recent meta-analysis reported that the AUC of PESI and sPESI for predicting all-cause mortality in APE ranged from 0.78 to 0.83 and was lower for predicting severe adverse events (0.64–0.68) [25]. Our findings suggest that combined radiomics–clinical models may outperform traditional prognostic tools, although this requires confirmation through prospective validation. Nevertheless, despite the promising performance of combined radiomics–clinical models, several practical barriers may hinder their integration into routine care. These include the need for high-performance computing infrastructure, the lack of standardized workflows across institutions, and limited interoperability with electronic health record (EHR) systems. Moreover, implementing radiomics in clinical environments requires user-friendly, automated tools that can operate within existing radiology information systems. Overcoming these barriers will require multidisciplinary collaboration among clinicians, radiologists, medical physicists, and data scientists, as well as regulatory frameworks that support standardization, validation, and certification of AI-based tools.

This review has several limitations. First, all included studies were retrospective and varied significantly in their methodology, including differences in imaging protocols, segmentation tools, and feature selection strategies. Second, sample sizes were often small, which may increase the risk of overfitting and reduce generalizability. For instance, Yang et al.’s study included only 74 patients, which limits the model’s robustness despite the reported high AUC in internal validation. In radiomics, where hundreds or thousands of features are extracted from medical images, small datasets increase the likelihood that the model will fit noise rather than true signal. This limitation may artificially inflate performance metrics in the training or internal test sets while performing poorly in external cohorts. Third, the outcome definitions were heterogeneous, and some studies lacked key clinical endpoints such as recurrence, quality of life, or long-term mortality. Fourth, none of the studies performed external validation in independent cohorts, limiting the ability to assess reproducibility. This methodological constraint is critical in radiomics, where models developed on big and homogeneous datasets may exhibit high apparent performance due to overfitting, but fail when applied to real-world populations with greater variability. External validation is essential to demonstrate generalizability, assess model calibration, and evaluate clinical utility. The lack of external validation in the included studies prevents meaningful assessment of robustness and hinders their translation into clinical practice. Future research should prioritize prospective multicenter designs with predefined external validation cohorts. Fifth, only two studies [16,18] reported a Radiomics Quality Score (RQS), which is a radiomics-specific tool for assessing methodological robustness. This limitation underscores the need for wider adoption of RQS in future radiomics research to enhance consistency and reproducibility.

## 5. Conclusions

Radiomics is a promising high-throughput method that may yield novel imaging biomarkers to improve the prediction of adverse outcomes in patients with APE. By enabling earlier and more precise therapeutic interventions, this approach has the potential to reduce mortality associated with APE significantly. However, the utility of radiomics-based strategies must be further validated in larger, prospective studies. Furthermore, expanding access to artificial intelligence tools and ensuring adequate training in resource-limited settings will be essential for their clinical implementation.

## Figures and Tables

**Table 1:** Clinical prediction rules for predicting 30-day mortality in patients with acute pulmonary embolism.

Pulmonary Embolism Severity Index (PESI)		
Predictors	Points	
Age (years)	Age	
Male sex	+10	
Cancer (previous or active)	+30	
Heart failure	+10	
Chronic lung disease	+10	
Pulse ≥ 110 beats per minute	+20	
Systolic blood pressure < 100 mmHg	+30	
Respiratory rate ≥ 30 breaths per minute	+20	
Temperature < 36 °C	+20	
Altered mental status	+60	
Arterial oxygen saturation < 90%	+20	
Risk classes	Risk stratification	Risk of 30-day mortality
Class I (≤65 points)	Very low risk	0.0–1.6%
Class II (66–85 points)	Low risk	1.7–3.5%
Class III (86–105 points)	Intermediate risk	3.2–7.1%
Class IV (106–125 points)	High risk	4.0–11.4%
Class V (>125 points)	Very high risk	10.0–24.5%
Simplified Pulmonary Embolism Severity Index (sPESI)		
Predictors	Points	
Age > 80 years	+1	
Cancer (previous or active)	+1	
Chronic lung disease	+1	
Pulse ≥ 110 beats per minute	+1	
Systolic blood pressure < 100 mmHg	+1	
Arterial oxygen saturation < 90%	+1	
Risk classes	Risk stratification	Risk of 30-day mortality
0 points	Low risk	1.1%
≥1 points	High risk	8.9%

**Table 2:** Characteristics of the included studies.

Author, Year	Target Population	Country	Study Design	Sample Size	Age, Mean ± SD	Female, n, %	Data Collection Period	Outcome PE Definition	Patients in the Training Set, n	Patients in the Validation Set, n
Zhou et al., 2018 [13]	Non-high- risk patients	China	Retrospective cohort	285	NR	NR	April 2013 to April 2017	Adverse outcomes in non-high-risk APE patients	170	115
Leonhardi et al., 2023 [14]	APE patients	Germany	Retrospective cohort	216	65 (17–99) *	116 (53.7)	2014 to 2019	Mortality, ICU admission, and sepsis-related organ failure	NR	NR
Yang et al., 2024 [15]	APE patients	China	Retrospective cohort	74	64.64 ± 10.73	34 (45.9)	December 2019 to August 2022	Mortality occurring within 30 days following APE or the requirement for mechanical ventilation, cardiopulmonary resuscitation, thrombolysis, vasopressor therapy, or catheter intervention	51	23
Gotta et al., 2024 [16]	APE patients	Germany	Retrospective cohort	131 (88 with central APE, 16 with peripheral APE, and 27 in the control group without APE)	64 ± 15	55 (42.0)	January 2015 to March 2022	Risk stratification and early death in APE	62	42
Gotta et al., 2024 [17]	APE patients	Germany	Retrospective cohort	131	64 ± 15	55 (42.0)	January 2015 to March 2022	Prediction of survival	79	52
Gotta et al., 2024 [18]	APE patients	Germany	Retrospective	136	63 ± 15	54 (39.7)	January 2015 to March 2022	Prediction of complicated courses requiring at least IMCU admission	81	55
Shahzadi et al., 2024 [19]	APE patients	Germany	Retrospective cohort	829	65 *	385 (46.4)	2005 to 2021	7- and 30-day all-cause mortality	580	249
Surov et al., 2024 [20]	APE patients	Germany	Retrospective cohort	284	64.5 ± 16.6	139 (48.9)	2015 to 2021	7-day and 30-day all-cause mortality	198	86
Wang et al., 2025 [21]	APE Patients	China	Retrospective cohort	321	64.2 ± 15.1	157 (48.9)	January 2015 to March 2022	30-day mortality and prolonged hospital stay (>10 days)	224	97

* Median (range). Abbreviations: APE, acute pulmonary embolism; ICU, intensive care unit; IMCU, intermediate care unit; NR, not reported; SD, standard deviation.

**Table 3:** CTPA acquisition protocols in the included studies.

Author, Year	CT Scanner	Slice Thickness	Tube Voltage (kVp)	Contrast Protocol	CT Scanner Protocol
Zhou et al., 2018 [13]	NR	NR	NR	NR	NR
Leonhardi et al., 2023 [14]	SOMATOM Force (Siemens)	1 mm	90/Sn150	Imeron 400, 80–100 mL at 5 mL/s, bolus tracking	SOMATOM Force (Siemens); DECT; 90/Sn150 kVp; 1 mm; Bv40 kernel
Yang et al., 2024 [15]	SOMATOM Force (Siemens)	1 mm	90/Sn150	Iopromide 370, 80 mL at 5 mL/s + 30 mL saline	SOMATOM Force (Siemens); DECT; 90/Sn150 kVp; 1 mm slice; kernel NR
Gotta et al., 2024 [16]	SOMATOM Force (Siemens)	1 mm	90/Sn150	Imeron 400, 100 mL at 5 mL/s, bolus tracking	SOMATOM Force (Siemens); DECT; 90/Sn150 kVp; 1 mm; Bv36 kernel
Gotta et al., 2024 [17]	SOMATOM Force (Siemens)	1 mm	90/Sn150	Imeron 400, 100 mL at 5 mL/s, bolus tracking	SOMATOM Force (Siemens); DECT; 90/Sn150 kVp; 1 mm; Bv36 kernel
Gotta et al., 2024 [18]	SOMATOM Force (Siemens)	1 mm	90/Sn150	Imeron 400, 80–120 mL at 5–6 mL/s	SOMATOM Force (Siemens); DECT; 90/Sn150 kVp; 1 mm slice; kernel NR
Shahzadi et al., 2024 [19]	SOMATOM Force (Siemens)	1 mm	100	Iopromide 370, 80 mL at 5 mL/s + 30 mL saline	SOMATOM Force (Siemens); 100 kVp; 1 mm slice; kernel NR
Surov et al., 2024 [20]	SOMATOM Definition AS + (Siemens)	1 mm	100–140 (modulated)	Accupaque 300 or Imeron 300, 60–150 mL at 3–4 mL/s, bolus tracking	SOMATOM Definition AS + (Siemens); 100–140 kVp (modulated); 1 mm slice thickness
Wang et al., 2025 [21]	SOMATOM Definition Flash (Siemens)	1 mm	100	Iopromide 370, 80 mL at 5 mL/s + 30 mL saline	SOMATOM Definition Flash (Siemens); 100–140 kVp (weight-adjusted); 1 mm slice thickness

Abbreviations: CT, computed tomography; CTPA, computed tomography pulmonary angiography; DECT, dual-energy computed tomography; kVp, kilovoltage peak; mL, milliliters; mm, millimeters; NR, not reported; s, seconds; Sn150, tin-filtered 150 kVp tube voltage.

**Table 4:** Performance of radiomic models in identifying patients with high risk of adverse outcomes.

Author, Year	Software Used for Feature Extraction	Anatomical Source of Radiomic Features	Radiomics Features Extracted, n	Models	Radiomics Features Used in Models	AUC (95% CI) of the Model in the Training Set	AUC (95% CI) of the Model in the Validation Set
Zhou et al., 2018 [13]	NR	Epicardial fat	NR	Radiomic model	RVD4-CH/LVD4-CH ratio and interventricular septum curvature positive	0.87	0.784
Leonhardi et al., 2023 [14]	MaZda (version 4.7)	Pulmonary embolus	279	Radiomic model	-S (5,5) Correlat and S (3,3) SumEntrp correlated with mortality -S (3,−3) AngScMom correlated with sepsis-related organ failure	NR	NR
Yang et al., 2024 [15]	PyRadiomics Python package (version 2.2.0) and D-Slicer	Pulmonary embolus	1037	Clinical model	Age and sex	0.778 (0.639–0.882)	0.833 (0.621–0.954)
				Radiomic model	RV/LV ≥ 1.0 and radiomics score	0.907 (0.792–0.970)	0.817 (0.601–0.945)
				Combined nomogram	Age, sex, RV/LV ≥ 1.0, and radiomics score	0.925 (0.816–0.980)	0.917 (0.724–0.990)
Gotta et al., 2024 [16]	PyRadiomics and 3D Slicer software (version 5.1.0–2022-05–20)	Pulmonary embolus	107	Radiomic model	Twelve features in the central PE group, seven features in the peripheral PE group, and 15 features in the group with all PE	0.91 in the central APE cohort	Central APE cohort: 0.86 (0.645–0.956) Peripheral APE cohort: 0.63 (0.38–0.869)
Gotta et al., 2024 [17]	PyRadiomics extension package was employed in the 3D Slicer software (version 5.1.0–2022–05-20)	Pulmonary embolus	107	Unadjusted radiomic model	Unadjusted radiomic model: voxel number, elongation, flatness, least axis length, major axis length, gray level non-uniformity, surface volume ratio, 10th percentile, root mean squared, energy, skewness, and total energy	NR	0.991 (0.979–1.000)
				Radiomic model adjusted by age			0.991 (0.979–1.000)
				Radiomic model adjusted by troponin			0.989 (0.973–1.000)
				Radiomic model adjusted by PESI score			0.991 (0.979–1.000)
Gotta et al., 2024 [18]	PyRadiomics extension package was employed in the 3D Slicer software (version 5.1.0–2022–05-20)	Pulmonary embolus	107	Unadjusted radiomic model	Elongation, flatness, and mesh volume	NR	0.63
				Adjusted radiomic model	Elongation, flatness, mesh volume, voxel volume, 90th percentile, energy, mean, median, and total energy	NR	0.58
Shahzadi et al., 2024 [19]	ImageJ software (version 1.53)	Skeletal muscle (T12) and intramuscular adipose tissue	234	sPESI score 7 days	Age > 80 years, cancer (previous or active), chronic lung disease, pulse ≥ 110 beats per minute, systolic blood pressure < 100 mmHg and arterial oxygen saturation < 90%	0.74 (0.66–0.82)	0.73 (0.67–0.79)
				sPESI score 30 days		0.72 (0.67–0.77)	0.74 (0.66–0.82)
				Radiomic SM 7 days	-stat_rms (root mean square) -morph_pca_elongation (morphological elongation) -szm_sze_2d_fbn_n24 (small zone emphasis in GLSZM)	0.71 (0.64–0.77)	0.56 (0.43–0.69)
				Radiomic SM 30 days		0.73 (0.67–0.78)	0.64 (0.53–0.74)
				Radiomic IMAT 7 days	-morph_comp_1 (morphological compactness) -stat_qcod (quantile coding) -ngl_glnu_d1_a0_2d_fbn_n24 (gray level non-uniformity in NGLDM)	0.70 (0.63–0.77)	0.62 (0.50–0.74)
				Radiomic IMAT 30 days		0.73 (0.67–0.79)	0.68 (0.57–0.78)
				Radiomic SM + IMAT 7 days	-stat_skew (skewness of intensity distribution) -szm_sze_2d_fbn_n24 (small zone emphasis in GLSZM) -morph_pca_elongation (morphological elongation)	0.74 (0.68–0.80)	0.57 (0.46–0.67)
				Radiomic SM + IMAT 30 days		0.77 (0.72–0.81)	0.70 (0.60–0.79)
Surov et al., 2024 [20]	PyRadiomics	Pulmonary embolus	107, plus two external validation cohorts of 169 and 186 patients	Logistic regression, random forest	First-order features (energy, kurtosis, and skewness), and texture features (GLRLM, GLSZM, and GLDM) from epicardial adipose tissue	7-day mortality: 0.724 (0.650–0.798) 30-day mortality: 0.776 (0.709–0.843)	7-day mortality: 0.750 (0.662–0.839) 30-day mortality: 0.721 (0.633–0.809)
Wang et al., 2025 [21]	3D-Slicer (PyRadiomics)	Pulmonary embolus	132	Clinical model	Four key features selected from GLRLM, GLCM, GLDM, and GLSZM matrices.	0.85 (0.78–0.92)	0.83 (0.74–0.91)
				Radiomics model		0.76 (0.67–0.84)	0.74 (0.64–0.84)
				Combined model (clinical plus radiomics)		0.89 (0.83–0.95)	0.901 (0.808–0.994)
				ML models (logistic regression, decision tree, random forest, and SVM)		0.91 (0.90–0.92)	No external validation
				DL models (ResNet-50 and VGG-19)		0.94 (0.93–0.95)	No external validation

Abbreviations: APE, acute pulmonary embolism; AUC, area under a receiver operating characteristic curve; CTPA, computed tomography pulmonary angiography; DECT, dual-energy computed tomography; DL, deep learning; GLRLM, Gray Level Run Length Matrix; GLCM, gray level co-occurrence matrix; GLDM, Gray Level Dependence Matrix; GLSZM, Gray Level Size Zone Matrix; IMAT, intramuscular adipose tissue; LV, left ventricular; LVD4-CH, left ventricular diameter in a four-chamber view; ML, machine learning; NR, not reported; PESI, Pulmonary Embolism Severity Index; ResNet-50, Residual Neural Network with 50 layers; RV, right ventricular; RVD4-CH, right ventricular diameter in a four-chamber view; SM, skeletal muscle; sPESI, Simplified Pulmonary Embolism Severity Index; SVM, support vector machine; VGG-19, Visual Geometry Group-19 (a type of convolutional neural network).

**Table 5:** Newcastle–Ottawa Scale **^†^** scores for the quality assessment of included studies.

Author, Year	Selection	Comparability	Outcome	Total Stars	Risk of Bias
Zhou et al., 2018 [13]	★★★	★★	★★	7	Low
Leonhardi et al., 2023 [14]	★★★	★★	★★★	8	Low
Yang et al., 2024 [15]	★★★	★★	★★★	8	Low
Gotta et al., 2024 [16]	★★★	★★	★★★	8	Low
Gotta et al., 2024 [17]	★★★	★★	★★★	8	Low
Gotta et al., 2024 [18]	★★★	★★	★★★	8	Low
Shahzadi et al., 2024 [19]	★★★	★★	★★	7	Low
Surov et al., 2024 [20]	★★★	★★	★★	7	Low
Wang et al., 2025 [21]	★★★	★★	★★★	8	Low

^†^ According to the Newcastle–Ottawa Scale, each ★ represents one fulfilled item within the domain: Selection (max ★★★) = representativeness of the cohort, selection of the non-exposed cohort, ascertainment of exposure, demonstration that outcome was not present at start; Comparability (max ★★) = control for the most important confounding factor, control for an additional factor; Outcome (max ★★★) = assessment of outcome, follow-up long enough for outcomes to occur, adequacy of follow-up of cohorts.

## Data Availability

Data is provided within the manuscript. The data of this study are available from the corresponding author and first author on reasonable request.

## Supplementary Materials

The following supporting information can be downloaded at https://www.mdpi.com/article/10.3390/diagnostics15162022/s1, Table S1: Outcome Definitions.

## Abbreviations

The following abbreviations are used in this manuscript:

AI	Artificial intelligence
APE	Acute pulmonary embolism
AUC	Area under a receiver operating characteristic curve
CTPA	Computed tomography pulmonary angiography
ML	Machine learning
NOS	Newcastle–Ottawa Scale
NPV	Negative predictive value
OR	OR
PESI	Pulmonary Embolism Severity Index
sPESI	Simplified Pulmonary Embolism Severity Index
PPV	Positive predictive value
VTE	Venous thromboembolism

## References

[1] Raskob G.E., Angchaisuksiri P., Blanco A.N., Buller H., Gallus A., Hunt B.J., Hylek E.M., Kakkar A., Konstantinides S.V., McCumber M. (2014). Thrombosis: A major contributor to global disease burden. Arter. Thromb. Vasc. Biol..

[2] Cohen A.T., Agnelli G., Anderson F.A., Arcelus J., Bergqvist D., Brecht J.G., Greer I.A., Heit J.A., Hutchinson J.L., Kakkar A.K. (2007). Venous Thromboembolism (VTE) in Europe. The number of VTE events and associated morbidity and mortality. Thromb. Haemost..

[3] Konstantinides S.V., Meyer G., Becattini C., Bueno H., Geersing G.J., Harjola V.-P., Huisman M.V., Humbert M., Jennings C.S., Jiménez D. (2020). 2019 ESC Guidelines for the diagnosis and management of acute pulmonary embolism developed in collaboration with the European Respiratory Society (ERS). Eur. Heart J..

[4] Gal G., Fine M.J., Roy P.-M., Sanchez O., Verschuren F., Cornuz J., Meyer G., Perrier A., Righini M., Aujesky D. (2008). Prospective validation of the Pulmonary Embolism Severity Index. Thromb. Haemost..

[5] Kohn C.G., Mearns E.S., Parker M.W., Hernandez A.V., Coleman C.I. (2015). Prognostic accuracy of clinical prediction rules for early post-pulmonary embolism all-cause mortality. Chest.

[6] Elias A., Mallett S., Daoud-Elias M., Poggi J.-N., Clarke M. (2016). Prognostic models in acute pulmonary embolism: A systematic review and meta-analysis. BMJ Open.

[7] Lankeit M., Jiménez D., Kostrubiec M., Dellas C., Hasenfuss G., Pruszczyk P., Konstantinides S. (2011). Predictive value of the high-sensitivity troponin T assay and the simplified Pulmonary Embolism Severity Index in hemodynamically stable patients with acute pulmonary embolism: A prospective validation study. Circulation.

[8] Santagata D., Donadini M.P., Ageno W. (2024). Use of artificial intelligence and radiomics for risk stratification in patients with pulmonary embolism: New tools for an old problem. Eur. J. Clin. Investig..

[9] Page M.J., Moher D., Bossuyt P.M., Boutron I., Hoffmann T.C., Mulrow C.D., Shamseer L., Tetzlaff J.M., Akl E.A., Brennan S.E. (2021). PRISMA 2020 explanation and elaboration: Updated guidance and exemplars for reporting systematic reviews. BMJ.

[10] Dekkers O.M., Vandenbroucke J.P., Cevallos M., Renehan A.G., Altman D.G., Egger M. (2019). COSMOS-E: Guidance on conducting systematic reviews and meta-analyses of observational studies of etiology. PLoS Med..

[11] Swets J.A. (1988). Measuring the accuracy of diagnostic systems. Science.

[12] Wells G., Shea B., O’Connell D., Robertson J., Peterson J., Welch V., Losos M., Tugwell P. The Newcastle-Ottawa Scale (NOS) for Assessing the Quality if Nonrandomized Studies in Meta-Analyses. http://www.ohri.ca/programs/clinical_epidemiology/oxford.htm.

[13] Zhou X., Hou G. (2018). A radiomics nomogram based on CT pulmonary angiographic data for predicting adverse outcomes in non-high-risk acute pulmonary embolism patients. Eur. Respir. J..

[14] Leonhardi J., Bailis N., Lerche M., Denecke T., Surov A., Meyer H.-J. (2023). Computed Tomography Embolus Texture Analysis as a Prognostic Marker of Acute Pulmonary Embolism. Angiology.

[15] Yang F., Chen R., Yang Y., Yang Z., Su Y., Ji M., Pang Z., Wang D. (2024). Computed tomography-based radiomics model to predict adverse clinical outcomes in acute pulmonary embolism. J. Thromb. Thrombolysis.

[16] Gotta J., Koch V., Geyer T., Martin S.S., Booz C., Mahmoudi S., Eichler K., Reschke P., D’ANgelo T., Klimek K. (2024). Imaging-based risk stratification of patients with pulmonary embolism based on dual-energy CT-derived radiomics. Eur. J. Clin. Investig..

[17] Gotta J., Gruenewald L.D., Martin S.S., Booz C., Mahmoudi S., Eichler K., Gruber-Rouh T., Biciusca T., Reschke P., Juergens L.-J. (2024). From pixels to prognosis: Imaging biomarkers for discrimination and outcome prediction of pulmonary embolism: Original Research Article. Emerg. Radiol..

[18] Gotta J., Gruenewald L.D., Geyer T., Eichler K., Martin S.S., Mahmoudi S., Booz C., Biciusca T., Reschke P., Juergens L.-J. (2024). Indicators for Hospitalization in Acute Pulmonary Embolism: Uncover the Association Between D-dimer Levels, Thrombus Volume and Radiomics. Acad. Radiol..

[19] Shahzadi I., Zwanenburg A., Frohwein L.J., Schramm D., Meyer H.J., Hinnerichs M., Moenninghoff C., Niehoff J.H., Kroeger J.R., Borggrefe J. (2024). Short-term mortality prediction in acute pulmonary embolism: Radiomics values of skeletal muscle and intramuscular adipose tissue. J. Cachexia Sarcopenia Muscle.

[20] Surov A., Zimmermann S., Hinnerichs M., Meyer H.-J., Aghayev A., Borggrefe J. (2024). Radiomics parameters of epicardial adipose tissue predict mortality in acute pulmonary embolism. Respir. Res..

[21] Wang D., Chen R., Wang W., Yang Y., Yu Y., Liu L., Yang F., Cui S. (2025). Prediction of short-term adverse clinical outcomes of acute pulmonary embolism using conventional machine learning and deep Learning based on CTPA images. J. Thromb. Thrombolysis.

[22] Jiménez D., Aujesky D., Moores L., Gómez V., Lobo J.L., Uresandi F., Otero R., Monreal M., Muriel A., Yusen R.D. (2010). Simplification of the pulmonary embolism severity index for prognostication in patients with acute symptomatic pulmonary embolism. Arch. Intern. Med..

[23] Soffer S., Klang E., Shimon O., Barash Y., Cahan N., Greenspana H., Konen E. (2021). Deep learning for pulmonary embolism detection on computed tomography pulmonary angiogram: A systematic review and meta-analysis. Sci. Rep..

[24] Su H., Han Z., Fu Y., Zhao D., Yu F., Heidari A.A., Zhang Y., Shou Y., Wu P., Chen H. (2022). Detection of pulmonary embolism severity using clinical characteristics, hematological indices, and machine learning techniques. Front. Neurosci..

[25] Zhou X.-Y., Ben S.-Q., Chen H.-L., Ni S.-S. (2012). The prognostic value of pulmonary embolism severity index in acute pulmonary embolism: A meta-analysis. Respir. Res..

