# Supplementary material for: Use of Radiomics to Predict Adverse Outcomes in Patients with Pulmonary Embolism: A Scoping Review of an Unresolved Clinical Challenge
Source: Diagnostics (Basel). 2025 Aug 12;15(16):2022. doi: 10.3390/diagnostics15162022 (PMC12385581; doi:10.3390/diagnostics15162022)
Supplement: Supplementary file 1 [file diagnostics-15-02022-s001.zip › diagnostics-3763296-supplementary.pdf]

Supplementary Table S1. Outcome Definitions.

Study (Author, year)	Outcome(s)	Time Frame	Composite	Detailed Definition
Zhou et al., 2018 [13]	Adverse outcomes	NR	Yes	Adverse outcomes in non-high-risk APE (not further specified)
Leonhardi et al., 2023 [14]	Mortality, ICU admission, sepsis-related organ failure	NR	Yes	Composite outcome including ICU admission and sepsis
Yang et al., 2024 [15]	Clinical deterioration or death	30-day	Yes	30-day mortality or need for ventilation, CPR, thrombolysis, vasopressors, or catheter
Gotta et al., 2024 [16]	Risk stratification/early death	NR	No	Risk category assignment per ESC (death considered as endpoint)
Gotta et al., 2024 [17]	Survival	NR	No	Survival (timing not specified)
Gotta et al., 2024 [18]	Complicated course	Hospital stay	Yes	Defined as IMCU admission or complications
Shahzadi et al., 2024 [19]	All-cause mortality	7-day and 30-day	No	7- and 30-day mortality separately assessed
Surov et al., 2024 [20]	All-cause mortality	7-day and 30-day	No	Standard mortality endpoints
Wang et al., 2025 [21]	Mortality and prolonged hospital stay	30-day	Yes	30-day mortality and length of stay >10 days

Abbreviations: APE: acute pulmonary embolism; CPR: cardiopulmonary resuscitation; ESC: European Society of Cardiology; ICU: intensive care unit; IMCU: intermediate care unit; NR: not reported.
